# Towards continuous brain monitoring: a pilot qualitative study of user experiences with in-ear EEG

**DOI:** 10.3389/fdgth.2026.1910034

**Published:** 2026-08-31

**Authors:** Chirag Haria, Benjamin H. L. Harris, Louis J. Koizia, Michael B. Fertleman

**Affiliations:** 1 Cutrale Perioperative and Ageing Group, Department of Bioengineering, Imperial College London, London, United Kingdom; 2 St. Catherine’s College, University of Oxford, Oxford, United Kingdom; 3 Department of Oncology, University of Oxford, Oxford, United Kingdom; 4 Institute for Liver and Digestive Health, Division of Medicine, University College London, London, United Kingdom; 5 Department of Psychosocial Rehabilitation, Medical University of Lodz, Lodz, Poland; 6 Collaborating Centre for Values-based Practice, St. Catherine’s College, Oxford, United Kingdom

**Keywords:** acceptability, continuous monitoring, electroencephalography, in-ear EEG, user experience, wearable neurotechnology

## Abstract

**Introduction:**

In-ear electroencephalography (EEG) is an emerging form of wearable neurotechnology that offers the potential for continuous and unobtrusive brain monitoring outside traditional clinical settings. While technical feasibility has been demonstrated across applications including seizure detection, sleep monitoring, and cognitive assessment, little is known about how users experience and perceive this technology. This pilot study aimed to explore user experiences of in-ear EEG and identify factors influencing its acceptability and adoption.

**Method:**

A qualitative study was conducted using semi-structured interviews with ten participants following exposure to an in-ear EEG device. Interviews were audio-recorded, transcribed verbatim, and analysed using an inductive approach guided by Braun and Clarke's reflexive thematic analysis.

**Results:**

Five interrelated themes were identified: embodied acceptability, pragmatic usability, reframing EEG from clinical procedure to personal technology, conditional adoption, and future integration into everyday life. Participants described the device as comfortable, unobtrusive, and comparable to familiar consumer technologies such as earbuds. Compared with conventional scalp EEG, in-ear EEG was viewed as more flexible, discreet, and compatible with everyday activities. However, willingness to adopt the technology remained conditional on demonstrating clinical accuracy comparable to traditional EEG systems. Participants also anticipated applications in long-term and ambulatory monitoring.

**Discussion:**

In-ear EEG was perceived as a highly acceptable and practical form of wearable brain monitoring. Adoption appeared to be facilitated by familiarity and integration into everyday life, but remained dependent on confidence in diagnostic performance. These findings highlight the importance of combining technical validation with user-centred design to support implementation of wearable neurotechnology and real-world monitoring of brain function.

## Introduction

The global population is ageing, and this demographic shift is accompanied by a rising burden of neurological disease, cognitive impairment, and acute brain dysfunction (1, 2). Continuous physiological monitoring is increasingly recognised as a key component of modern clinical care, enabling earlier detection of deterioration and more timely intervention (3), yet the brain remains comparatively under-monitored, with routine tracking focused on parameters such as heart rate, oxygen saturation, and blood pressure rather than real-time cerebral function. This creates a critical gap, particularly for conditions characterised by fluctuating or subclinical changes, such as delirium and non-convulsive seizures, where timely detection may alter outcomes.

Electroencephalography (EEG) remains the gold standard for measuring brain activity, offering high temporal resolution and direct insight into neuronal function (4). However, conventional scalp EEG is resource-intensive, requiring specialised equipment, trained personnel, and controlled environments. As a result, its use is typically episodic rather than continuous, limiting its ability to capture dynamic neurological changes over time and reducing accessibility in routine care settings.

Recent advances in wearable technology have led to the development of “earables”, devices designed to monitor physiological signals from within or around the ear (5). In-ear EEG represents a promising evolution of this approach, enabling the recording of brain activity through discreet, portable, and potentially more user-friendly devices (5). Compared with traditional scalp-based systems, in-ear EEG offers several theoretical advantages, including improved comfort, reduced obtrusiveness, and the potential for extended or continuous monitoring in both clinical and community environments (6). Early studies have demonstrated feasibility across a range of applications, including seizure detection, sleep monitoring, and cognitive assessment, suggesting that this technology may support more accessible and scalable neurophysiological monitoring (7, 8).

Despite these technological advances, the translation of in-ear EEG into real-world use depends not only on signal quality and diagnostic performance but also on user experience. Wearable devices must be acceptable, comfortable, and practical for sustained use, particularly in populations who may have sensory sensitivities, cognitive impairment, or physical limitations. Factors such as device comfort, ease of use, perceived stigma, and integration into daily life may significantly influence adherence and, consequently, data quality and clinical utility. However, to date, research in this field has largely focused on technical validation and performance metrics, with comparatively limited attention given to how users experience and interact with these devices.

Understanding user perspectives is particularly important in the context of emerging neurotechnology, where successful implementation relies on alignment between technological capability and human factors. Qualitative methods offer a valuable approach to exploring these dimensions, enabling in-depth examination of how individuals perceive, tolerate, and make sense of novel monitoring tools. Such insights can inform device design, clinical workflows, and implementation strategies, ultimately supporting more effective and patient-centred adoption.

This study therefore aims to explore user experiences of in-ear EEG using an inductive qualitative approach guided by reflexive thematic analysis (9). By examining acceptability, usability, and perceived role in both clinical and everyday contexts, this work seeks to address an important gap in the literature and contribute to the development of wearable neurotechnology that is not only technically robust but also aligned with the needs and preferences of its users.

## Material and methods

### Data collection

The project was registered as a Quality Improvement Project within Imperial College Healthcare NHS Trust. Informed consent was obtained before each interview. Semi-structured interviews were conducted on Microsoft Teams, lasting approximately 30–45 minutes. Interviews were conducted by Chirag Haria (specialist registrar in geriatrics). Efforts were made to maintain a conversational, non-clinical interview environment, emphasising participants’ experiences and perspectives. Interviews were audio-recorded, transcribed verbatim and anonymised during transcription. Field notes documented context and non-verbal observations. The semi-structured interview guide is provided in Supplementary File S1.

### In-ear EEG device

**Figure 1:**
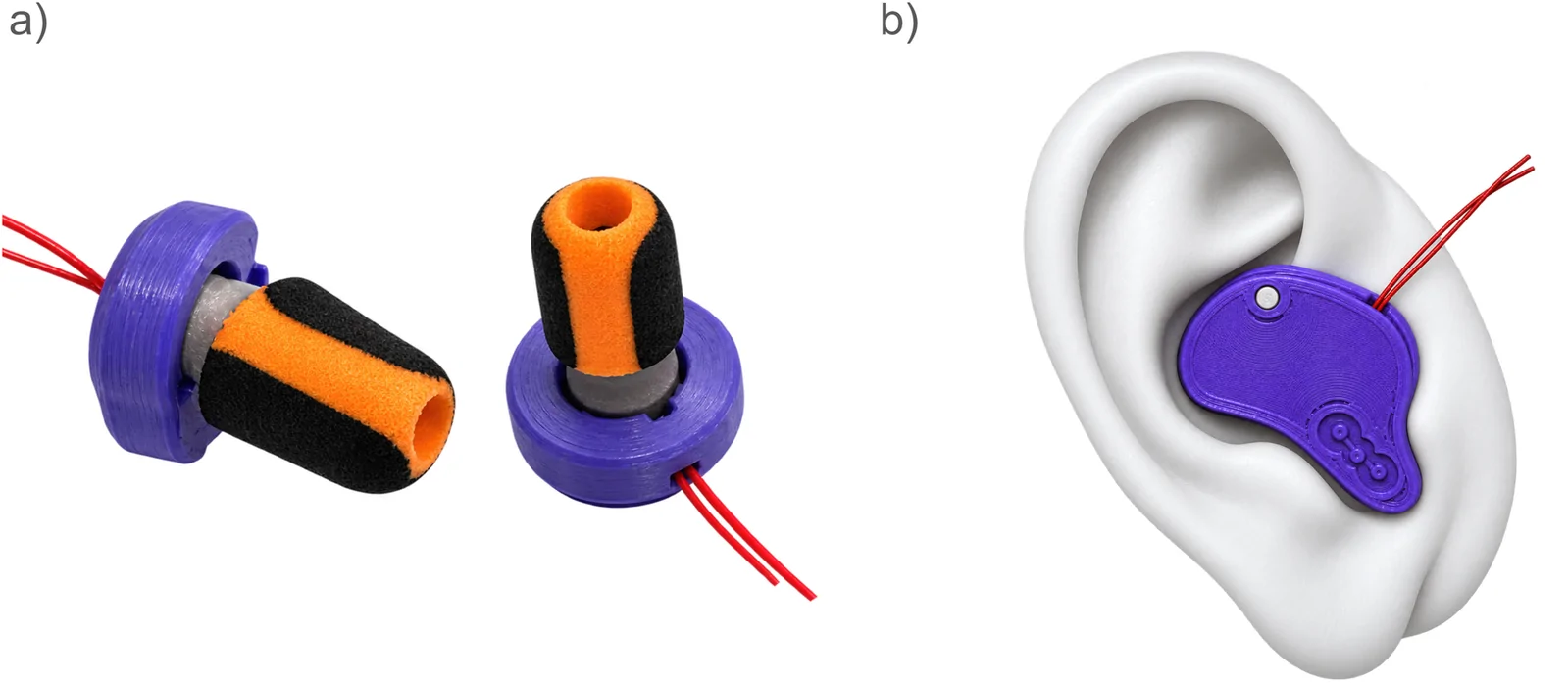
In-ear EEG device. **(a)** The in-ear EEG device consists of a three-dimensional soft rubber chassis that sits within the concha for stability and conductive elastomer-coated hearing protection earbuds (3M 1100; 3M, St. Paul, MN, USA) positioned within the external auditory canal. Each earbud incorporates two distinct electrodes, with individual wire leads exiting laterally through the rubber chassis. **(b)** The assembled in-ear EEG device in situ, illustrating its low-profile design and anatomical fit within the concha and external auditory canal.

The in-ear EEG sensor is a biopotential sensing device designed to sit within the concha and external auditory canal. The sensing element comprises a conductive elastomer, a novel electroactive composite coated onto commercially available hearing protection earbuds (3M 1100; 3M, St. Paul, MN, USA). The earbuds are mounted on a soft rubber chassis that provides stability within the concha. Each earbud incorporates two distinct recording electrodes positioned within the external auditory canal, with individual wire leads exiting laterally through the chassis, providing four recording electrodes in total. Unlike conventional scalp EEG, which typically uses multiple electrodes distributed across the scalp, this compact configuration enables unobtrusive recording from within and around the ear. The device is illustrated in Figure 1.

### Analysis

The pilot study adopted an exploratory qualitative design and did not employ a predefined theoretical framework as in previous studies (10). Instead, an inductive approach guided by Braun and Clarke's reflexive thematic analysis was used (9). Reflexive thematic analysis was chosen because it allows flexible exploration of participants’ experiences and meanings without imposing predefined theoretical assumptions. Initial open coding was conducted line by line, with codes iteratively organised into preliminary categories and interpretively developed into overarching themes. Two researchers independently coded all transcripts, with discrepancies resolved through discussion and consensus. The researchers engaged in iterative discussion throughout the analytical process to reflect on interpretations and refine themes. NVivo 14 facilitated systematic data organisation and supported the iterative refinement of themes. Participant checking (member checking) was not undertaken. Analytical rigour was supported through independent coding and iterative discussion during theme development.

### Ethical approval

As a registered Quality Improvement Project, formal NHS Research Ethics Committee review was not required. All participants provided informed consent. Data were anonymised and handled securely in accordance with institutional governance procedures.

## Results

Ten participants were included in the analysis (six men, four women, mean age 36 years). Reflexive thematic analysis generated five interrelated themes: embodied acceptability, pragmatic usability, reframing EEG from clinical procedure to personal technology, conditional adoption, and future integration into everyday life.

### Embodied acceptability: familiar, tolerable, and minimally intrusive

Participants consistently described the in-ear EEG device as comfortable and unobtrusive. Importantly, comfort was not framed purely in physical terms, but in relation to familiarity. The device was frequently interpreted through the lens of everyday consumer technologies, particularly earbuds, which appeared to reduce its perceived novelty and medical character.

- “It seemed like I was wearing earbuds.” (Participant 1, 36-year-old man)
- “It was comfortable enough for me to lay down and to be relaxed” (Participant 6, 30-year-old woman)
- “Comfort wise it was fine. I'd say occasionally it felt like you could feel the sort of the earplug kind of moving a little. But that was all really, it was fine.” (Participant 5, 32-year-old woman)

This sense of normality facilitated rapid habituation. Although participants initially noticed the device, this awareness did not translate into discomfort or distraction. Instead, it became part of the background of their experience. The stability of the device further reinforced this acceptability, allowing participants to remain largely unaware of it during use. Participants generally found the device comfortable once correctly positioned, although one participant emphasised that achieving a secure fit was an important determinant of comfort and acceptability.

- “I was aware that it was in my ear…there were no concerns that it would do any damage.” (Participant 1, 36-year-old man)
- “I fell asleep. I think I fell asleep most of the time” (Participant 10, 34-year-old man)
- “Once it settled, it was absolutely fine.” (Participant 3, 45-year-old woman)

Together, these accounts suggest that in-ear EEG achieves a high degree of embodied acceptability by aligning with familiar sensory and technological experiences. This was reflected in participants’ perceived comfort ratings, with the in-ear EEG receiving a median score of 8/10 (mean 8.1 ± 0.9) compared with 6.5/10 (mean 6.6 ± 1.8) for conventional scalp EEG. Seven participants (70%) assigned a higher comfort score to the in-ear EEG than scalp EEG, one (10%) rated both devices equally, and two (20%) reported greater comfort with the scalp EEG.

### Pragmatic usability: apparent simplicity and underlying technical work

Participants widely described the device as straightforward and easy to use, particularly when compared to conventional EEG systems. This perception of simplicity was often expressed confidently.

- “It wasn't very difficult at all… quite straightforward.” (Participant 9, 34-year-old woman)
- “Uh, it was pretty straightforward. It's pretty simple.” (Participant 2, 40-year-old man)
- “Well, so, It's not too difficult, it's just like wearing normal ear pieces.” (Participant 8, 35-year-old man)

However, closer examination revealed that achieving optimal performance required small but meaningful adjustments. Participants described the need to actively engage with the device to ensure good signal quality. They demonstrated an emerging understanding of the relationship between their behaviour and signal quality, particularly in relation to jaw movement and speech.

- “It did need a little bit of water to make sure that the contact was better.” (Participant 1, 36-year-old man)
- “I was anxious at the start that they would fall out if I did anything too vigorously, but basically talking, opening my mouth and my jaw.” (Participant 4, 39-year-old man)

These findings suggest that while the device is perceived as simple, its effective use involves a degree of ongoing interaction and adaptation, highlighting a tension between intuitive design and technical sensitivity. Furthermore, all participants (100%) described the device as straightforward to use, although several highlighted the need for minor adjustments to optimise signal quality.

### Reframing EEG: from clinical procedure to personal technology

Participants consistently contrasted in-ear EEG with traditional scalp EEG, using this comparison to highlight differences in complexity, mobility, and independence. Scalp EEG was described as requiring precise placement, specialist input, and additional materials. It was also perceived as restrictive in terms of movement and practicality.

- “Whereas the scalp, it's not uncomfortable. But you know it's there and obviously there's a number of leads and you know, I can't, I can't say to you that it was, it was nice. It wasn't.” (Participant 4, 39-year-old man)
- “I was not sitting there as long waiting for things to be applied.” (Participant 5, 32-year-old woman)

In contrast, in-ear EEG was associated with flexibility and autonomy, allowing participants to imagine more dynamic and real-world use cases. Through these comparisons, participants effectively reinterpreted EEG as a modality that could extend beyond the clinic into everyday environments, aligning it with the broader category of wearable technologies.

### Conditional adoption: accuracy as the determining factor

Despite overwhelmingly positive experiences in terms of comfort and usability, participants consistently emphasised that their willingness to adopt in-ear EEG would depend on its accuracy and reliability. Initial responses reflected clear preference and openness.

- “Much user friendly option in comparison to the scalp.” (Participant 4, 39-year-old man)
- “I can easily tolerate it.” (Participant 9, 34-year-old woman)

However, this preference was explicitly conditional on performance. Nevertheless, all participants (100%) indicated that they would be willing to use the in-ear EEG again if clinically indicated. These accounts indicate that while experiential factors strongly favour in-ear EEG, clinical validity remains the ultimate determinant of acceptance.

- “We don't know how much it records and whether it records as accurately the scalp. But if the device was, you know, found to be accurate and gave as good outcome as the scalp, then I'll probably prefer to wear the ear EEG.” (Participant 1, 36-year-old man)
- “If the recordings would be equally accurate, then I would choose the in-ear EEG.” (Participant 7, 33-year-old man)

### Future integration: continuous and real-world monitoring

Participants frequently projected the potential of in-ear EEG into future use scenarios, particularly in relation to long-term and ambulatory monitoring. The device was seen as well suited to contexts requiring extended recording and mobility.

- “I guess in situations where longer reporting is needed, and you need the subject to be more mobile.” (Participant 1, 36-year-old man)
- “I think there's quite a lot of opportunities to use it so I think the example of times when you needed to have a longer recording.” (Participant 4, 39-year-old man)

Participants also anticipated further improvements in portability and usability, suggesting a trajectory toward seamless integration into daily life. These forward-looking reflections position in-ear EEG within a broader shift toward continuous, decentralised health monitoring, consistent with the evolution of wearable technologies.

- “Yeah, really comfortable, I mean, it already looks like you're wearing earphones. If the final product was made to look more like earphones, in my opinion, I think anyone would be comfortable wearing that around.” (Participant 7, 33-year-old man)

Across themes, participants evaluated in-ear EEG less as a conventional medical device and more as a familiar wearable technology. Comfort, ease of use and perceived portability facilitated acceptance, while comparisons with scalp EEG encouraged participants to reconceptualise EEG as a technology capable of supporting autonomous, real-world monitoring. Nevertheless, enthusiasm for adoption remained contingent upon confidence that these practical advantages would not compromise diagnostic accuracy.

## Discussion

This study provides qualitative insights into user experiences of in-ear EEG. Overall, participants described the device as highly acceptable, easy to use, and compatible with everyday activity, while emphasising that its ultimate value depends on clinical accuracy. These findings highlight that the successful implementation of wearable neurotechnology depends not only on technical performance but also on how devices are experienced, interpreted, and integrated into daily life. Together, these findings suggest that adoption of wearable neurotechnology is a process of normalisation, in which unfamiliar medical devices become acceptable through alignment with everyday experiences (11).

A central finding of this study is that acceptability was not experienced purely as a physical property, but as a function of familiarity. Participants consistently interpreted the device through the lens of everyday consumer technologies, particularly earphones, which appeared to reduce its perceived novelty and medical character. This process of normalisation facilitated rapid habituation, with the device becoming part of the background of experience rather than a persistent source of awareness. In this context, in-ear EEG may benefit from occupying a conceptual space closer to personal technology than medical equipment.

Alongside this, participants described the device as straightforward and easy to use, reinforcing its potential for deployment beyond specialist settings. However, our findings also reveal a more nuanced picture: while usability was perceived as intuitive, effective use required small but meaningful adjustments, including attention to fit, movement, and signal contact. This tension between apparent simplicity and underlying technical sensitivity is consistent with emerging work in wearable biosensing (12, 13), where user interaction remains critical to data quality. These findings suggest that successful implementation will depend not only on device design but also on clear guidance, training, and user support.

Participants consistently contrasted in-ear EEG with conventional scalp EEG, reframing EEG from a complex, clinician-dependent procedure into a more flexible and personal technology. This shift has important implications. Traditional EEG is often episodic, resource-intensive, and poorly tolerated, limiting its ability to capture dynamic neurological changes over time. In contrast, participants readily imagined in-ear EEG as enabling longer-term, mobile, and real-world monitoring. Examples could include sleep monitoring, as in previous studies (14). This aligns with a broader shift in medicine towards continuous physiological monitoring and decentralised care, in which data are collected longitudinally rather than at isolated time points.

Importantly, however, adoption was explicitly conditional. Despite positive experiences of comfort and usability, participants consistently emphasised that they would only accept in-ear EEG if it demonstrated accuracy comparable to conventional systems. This reflects a central principle in digital health: while user experience enables engagement, clinical validity determines trust. Our findings therefore reinforce that technical validation and user-centred design must evolve in parallel if wearable neurotechnology is to achieve meaningful clinical impact.

These findings should be interpreted within the broader clinical context of neurological care in older adults. Syndromes such as delirium, non-convulsive seizures, and post-operative encephalopathy are characterised by fluctuation and are frequently under-recognised in routine care. Current approaches rely on intermittent assessment and limited access to EEG, meaning that clinically important changes may go undetected. In this context, the acceptability and tolerability demonstrated in our study are particularly significant. If in-ear EEG can provide continuous, unobtrusive monitoring, it may offer a means of observing the brain in real time, capturing dynamic changes that are currently missed. This represents a potential shift from reactive investigation to proactive monitoring.

Our findings also complement existing technical literature, which has demonstrated the feasibility of in-ear EEG for applications including seizure detection, sleep monitoring, and cognitive assessment (14–16). Consistent with prior work, participants recognised the potential for longer-term and ambulatory monitoring. However, our study extends this literature by demonstrating how these possibilities are interpreted by users themselves, highlighting the importance of familiarity, autonomy, and conditional trust in shaping adoption.

Several limitations should be acknowledged. This was a single-centre pilot study involving a relatively small sample of healthy adults with no co-morbidities, which may limit generalisability to other populations or settings. Participants were interviewed in a controlled clinical environment after short-term exposure to the device; experiences during prolonged use in community settings may differ. Furthermore, while in-ear EEG offers advantages in comfort and wearability, its restricted electrode location provides more limited spatial coverage than conventional scalp EEG and may reduce sensitivity to activity arising from deep brain structures or cortical regions distant from the ear. Consequently, in-ear EEG should currently be considered a complementary technology rather than a replacement for conventional scalp EEG until further technical validation is available. In addition, as with all qualitative research, findings are interpretive and shaped by the context of data collection and analysis. However, the use of reflexive thematic analysis and iterative coding supports the depth and credibility of the findings.

## Future directions

Future research should focus on evaluating in-ear EEG in real-world conditions over extended periods, including in populations with cognitive impairment or neurological disease. Head-to-head comparisons with conventional scalp EEG across a range of neurological conditions will be essential to define the diagnostic strengths and limitations of in-ear EEG and determine the clinical contexts in which it can be used most effectively. Advances in electrode materials, signal processing, and machine learning may improve data quality and interpretation. Equally important will be the development of implementation strategies that integrate wearable neurotechnology into clinical workflows, ensuring that data generated are actionable and clinically meaningful. Future research should also explore integration of in-ear EEG with complementary physiological and molecular biomarkers. Just as new assays and multimodal biomarker strategies have improved diagnostic sensitivity and specificity in other areas of medicine (17–19), combining continuous electrophysiological monitoring with circulating biomarkers of neuronal injury, neuroinflammation, or physiological stress, such as cytokines (20, 21), may provide a more comprehensive assessment of brain health than either modality alone.

## Conclusion

In conclusion, in-ear EEG represents a promising step towards continuous, user-centred brain monitoring. This study demonstrates that such devices are not only technically feasible but also acceptable and meaningful to users when they align with familiar experiences and everyday practices. However, their adoption will ultimately depend on demonstrating clinical accuracy and value. More broadly, in-ear EEG reflects a shift from observing the brain in controlled environments to monitoring it in real life. The challenge now is not only to refine the technology, but to ensure that it is implemented in a way that is both clinically effective and responsive to the needs of those who use it.

## Data Availability

The raw data supporting the conclusions of this article will be made available by the authors, without undue reservation.

## Supplementary material

SUPPLEMENTARY FILE 1 Semi-structured interview guide. Semi-structured interview questions used to explore participants’ experiences of using the in-ear EEG device, including comfort, usability, perceived safety, comparison with conventional scalp EEG, and potential future applications.
