# Supplementary material for: Towards continuous brain monitoring: a pilot qualitative study of user experiences with in-ear EEG
Source: Front Digit Health. 2026 Aug 31;8:1910034. doi: 10.3389/fdgth.2026.1910034 (PMC13572655; doi:10.3389/fdgth.2026.1910034)
Supplement: Supplementary file 1 [file Supplementaryfile1.docx]

1. Before wearing the in-ear EEG, what were your expectations?
2. Can you describe your overall experience wearing the in-ear EEG?
3. How did this compare to wearing the scalp EEG?
4. How comfortable did you find the in-ear EEG while it was in place?
5. Did you experience any unusual sensations?
6. Compared with the scalp EEG, which device felt more physically comfortable and why?
7. Did you have concerns about the safety of the in-ear EEG?
8. Overall, on a scale of 0 to 10, where 10 is the most comfortable, what would you rate the comfort of the in-ear EEG?
9. And the scalp EEG?
10. How easy or difficult was it to put on and remove the in-ear EEG?
11. How did this compare with the setup and removal of the scalp EEG?
12. How well did the in-ear EEG fit in your ear and how stable did it feel?
13. Did the in-ear EEG affect you in any way?
14. How aware were you of wearing the in-ear EEG?
15. How, if at all, did wearing the device affect what you were doing or how you felt during the session?
16. Overall, which device did you find easier to tolerate and why?
17. Would you have been comfortable wearing the in-ear EEG for a longer period of time?
18. Would you be willing to use the in-ear EEG again?
19. If you had to choose one device for future EEG recordings, which would you prefer and why?
20. In what situations do you think the in-ear EEG would be more suitable than scalp EEG?
21. How comfortable would you feel wearing the in-ear EEG outside the laboratory?
22. What do you like most about the in-ear EEG?
23. What was the least acceptable or most problematic aspect?
24. If you could change one thing about the in-ear EEG, what would it be?
25. Is there anything about your experience that we have not covered that you would like to talk about?

**Supplementary Material S1.** Semi-structured interview guide

Semi-structured interview questions used to explore participants' experiences of using the in-ear EEG device, including comfort, usability, perceived safety, comparison with conventional scalp EEG, and potential future applications.
